# Functional analysis of the interferon-stimulated response element of porcine circovirus type 2 and its role during viral replication *in vitro* and *in vivo*

**DOI:** 10.1186/1743-422X-9-152

**Published:** 2012-08-07

**Authors:** Jinyan Gu, Yu Zhang, Xue Lian, Hailiang Sun, Jingman Wang, Weiting Liu, Gang Meng, Peng Li, Dan Zhu, Yuexin Jin, Ruibing Cao

**Affiliations:** 1 Key Laboratory of Animal Diseases Diagnostic and Immunology, Ministry of Agriculture, College of Veterinary Medicine, Nanjing Agriculture University, Tong Wei Road 6#, Nanjing 210095, Jiangsu, China

**Keywords:** PCV2, P*rep*, ISRE, IFN-α, Replication, Regulation

## Abstract

**Background:**

Porcine circovirus type 2 (PCV2) is associated with post-weaning multi-systemic wasting syndrome (PMWS) in young weaned pigs. Immune stimulation was found to activate the replication of PCV2 and exacerbate the clinical outcome of the infection. Proper amount of interferon-α (IFN-α) is able to enhance PCV2 infection and production in Porcine kidney-15 (PK-15) cells when administered after inoculation.

**Methods:**

In the present study, luciferase reporter assays, construction of mutant viruses, Analysis the replication efficiency and the response to IFN-α treatment in PK-15 cells and animal experiments were carried out to analyze the function of interferon-stimulated response element (ISRE) of PCV2 and its role during viral replication *in vitro* and *in vivo*.

**Results:**

A functional viral ISRE sequence, 5′-CTGAAAACGAAAGA-3′, was identified in *Rep* gene promoter (P*rep*) of PCV2. PCV2 *Prep* is composed of two mini promoters, the proximal one span the sequence +1 to -106, containing an ISRE while the distal mini promoter is composed of three tandem GC box like sites locate at -85 to -194. It was demonstrated that viral ISRE is necessary for porcine IFN-α initiated luciferase expression enhancement and it plays an important role in affecting the replication efficiency of PCV2 *in vivo* and *in vitro*.

**Conclusions:**

These findings provide a theoretical basis for the Phenomenon of immunostimulation is able to enhance PCV2 infection, and improve the understanding of the complicated mechanisms involved in the host and pathogen interactions of PCV2.

## Background

PCV2 was identified as an essential causative agent of post-weaning multi-system wasting-syndrome (PMWS) [1]. PMWS has been observed in virtually all regions of the world involved in intensive pig production. The disease most commonly affects pigs between the ages of 5–18 weeks, clinical signs include a marked increase in the mortality rate, wasting, generalized enlargement of lymph nodes, respiratory signs, and occasionally pallor, jaundice and diarrhea [2].

The PCV2 is a small single circular ambisense DNA virus whose replication occurs in the nuclei of infected cells. With its limited number of viral proteins, PCV2 is not well equipped to modify the intracellular conditions in favor of its own needs for replication, However, relatively simple viruses, such as PCV2, might profit from specific conditions in the host that have been induced by other infectious agents [3].

It is generally believed that immunostimulation either by vaccination or secondary viral infection plays a role in the occurrence of PMWS [4-7]. However, the exact role of immunostimulation in the progression to clinical PMWS is still unknown.

Previous study showed that type I and type II interferons (IFNs) are able to enhance PCV2 infection and production in PK-15 and 3D4/31 cells. Porcine IFN-α enhance PCV2 infection by 529% in PK-15 cells and by 308% in 3D4/31 cells when it was added after inoculation [3]. It is a very important finding in understanding the interaction of circoviruses with their host cells and also in view of the pathogenesis and reproduction of PMWS in PCV2-infected pigs.

After binding to its host cell receptor, Type I IFN (IFN-a and IFN-β) induces the formation of a heterotrimetric transcription factor complex, interferon-stimulated gene factor 3 (ISGF3), which consists of signal transducers and activators of transcription 1 and 2 (STAT1 and STAT2) and p48 (IRF-9). ISGF3 translocates into the nucleus and binds to the ISRE in the promoter of a variety of interferon-inducible genes, and transactivates their expression [8]. Interferon regulatory factors (IRF) are another important factor induced by type I and II IFNs. Since the sequence of IRF binding site termed IRF-E overlaps with ISRE, IRF also binds to ISRE sequence and activates the interferon-inducible gene transcription, but in part, functionally different [9].

Previous studies have demonstrated the presence of ISRE like sequence in several viral genomes, such as the Epstein-Barr virus [10,11], the bovine leukemia virus [12], the human immunodeficiency virus type 1 [13] and the hepatitis B virus [14].It was shown that the PCV1 P*rep* contains an ISRE (nts. 742–753), indicating putative regulation by cytokines [15]. PCV2 also contains an ISRE like sequence 5′-CTGAAAACGAAAGA-3′ in the PCV2 *Rep* promoter region plays a role in PCV2’s response to IFN, But the biological function is altered when the ISRE sequence is removed from the context of the whole viral genome [16].

In the present study, luciferase reporter assay and nucleotide substitution mutagenesis were conducted to investigate whether the ISRE sequence in the PCV2 *Rep* promoter region is a functional element in and out of the genome and whether ISRE Mutation affects replication efficiency *in vivo* and *in vitro*. The results demonstrated that PCV2 ISRE sequence not only plays a role in the whole viral genome *in vivo* and *in vitro* but also work only in the *Rep* promoter, even individual. These findings are somewhat different from S. Ramamoorthy’s [16].

## Results

### The PCV2 ISRE sequence demonstrates biological function individually

**Figure 1:**
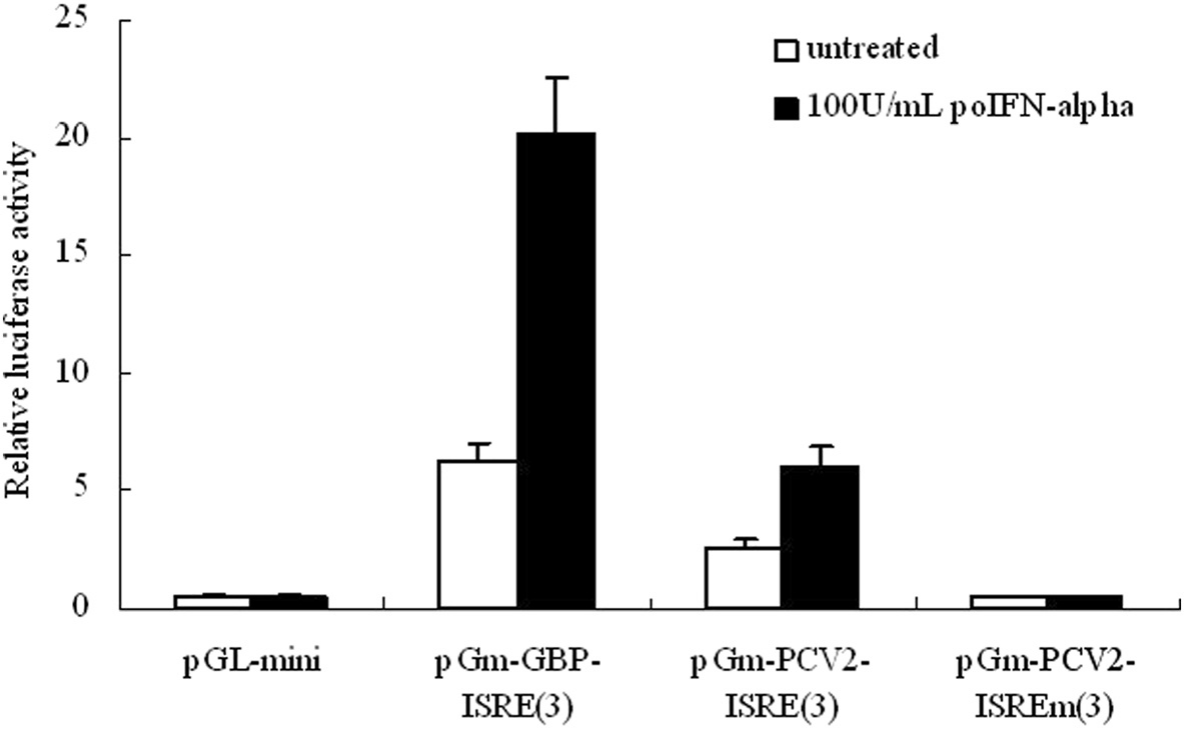
**Function analysis of the PCV2 ISRE in the context of an enhancer test vector.** The pGm-PCV2-ISRE(3), pGm-PCV2-ISREm(3) and pGm-GPB-ISRE(3) constructs contain the PCV2 ISRE, mutant PCV2 ISRE and GBP2 promoter ISRE double-stranded oligonucleotides cloned into the minimal promoter construct pGL-miniP, respectively. The number of copies of the oligonucleotide in the constructs is shown in parenthese in the construct name. Luciferase activity was measured in extracts of transfected cells treated with porcine IFN-α, and compared with untreated cells. pRL-TK was included in every transfection mixture to control for variations in transfection efficiencies. The level of Renila luciferase in each sample was used to normalize firefly luciferase activity. The final concentration of porcine IFN-α was 100 U/mL. Experiments were carried out independently at least 3 times and the results are expressed as means ± SD.

There is a ISRE like sequence 5′-CTGAAAACGAAAGA-3′ locates at nt 1737–1751 in the PCV2 *Rep* promoter which is similar to the ISRE sequence in interferon-inducible gene promoters (Table 1). To determine whether the ISRE sequence in PCV2 *Rep* promoter is individual response to IFN-α treatment, the synthetic multiple viral ISRE was examined in the context of an enhancer test vector pGL-miniP. pGL-miniP was constructed by substituted the SV40 promoter of pGL3-Promoter with HSV-1 TATA-like promoter (P_TAL_). The level of luciferase transcription from the construct pGm-PCV2-ISRE(3) which contains three copies of the PCV2 ISRE sequence, increased 4-fold compared to pGL-miniP and 10-fold in the presence of 100U/mL porcine IFN-α treatment. Mutation of the viral ISRE sequence in the construct pGm-PCV2-ISRE mut(3) completely inhibited enhancement of transcription and IFN-α treatment also failed to greatly modulated transcription (Figure 1). Enhancement of transcription by porcine IFN-α treatment demonstrates that the ISRE like sequence in PCV2 *Rep* promoter region can act as an interferon-stimulated response element individually.

**Table 1:** Sequence similarity between ISRE sequence in the interferon inducible gene promoters and ISRE-like sequence in virus promoter

**Gene**	**Sequence**	**Ref.**
Mx1 (porcine)	GG**GAAA**C**GAAA**CT	Tungtrakoolsub, 2007
Mx2 (porcine)	AG**GAAA**T**GAAA**CT	Tungtrakoolsub, 2007
GPB (porcine)	AT**GAAA**CT**GAAA**GC	Ma, 2008
HIV-1 LTR	TT**GAAA**GC**GAAA**GG**GAAA**CC	Battistini,2002
HBV enhancer-1	GA**GAAA**GT**GAAA**GC	Alcantara, 2002
EBV Qp	GC**GAAA**AC**GAAA**GT	Zhang, 1997
PCV2 P*rep*	CT**GAAA**AC**GAAA**GA	-

The pGm-GBP-ISRE(3) constructs was proved to have similar, but somewhat greater, responsiveness to porcine IFN-α treatment, confirming that the PCV2 *Rep* promoter ISRE sequence can act as an interferon-stimulated response element in a manner similar to previously characterized ISRE-containing promoter [17].

### Characterization of the ISRE containing *Rep* gene promoter

Promoter P*rep* overlaps the intergenic region and the origin of replication. Sequence analysis showed that PCV2 *Rep* promoter does not contain any recognizable TATA or CAAT elements. However, it contains several elements that may influence its activity. Two AP1 boxes (nt −252 to −255 and −267 to −280), one AP2 site (nt −227 to −222) and four SP1 sites (nt −122 to −127, −158 to −163, −178 to −183, and −233 to −238), are located upstream of the ISRE (nt −67 to −81).

**Figure 2:**
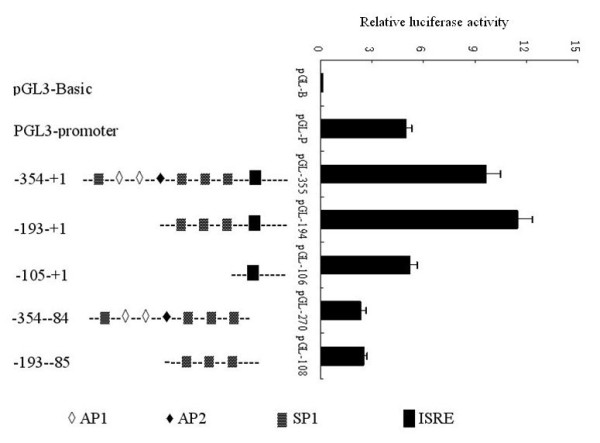
** Promoter activities of porcine* Rep*gene with partial deleted constructs.** The schema on the left shows a representation of the partial deleted constructs of porcine *Rep* promoters, and that on the right shows the promoter activities assayed in PK-15 cells. Experiments were carried out independently at least 3 times and the results are expressed as means ± SD.

To character the P*rep*, fragments containing the putative promoters were cloned into plasmid pGL3-basic in front of a promoterless *luc* gene. We assumed that P*rep* was located immediately upstream of the *Rep* translation start position at nt 51. The resultant pGL-355 plasmids were investigated for promoter activity using the Dual-Luc assay. Compared with the late SV40 promoter in plasmid pGL3-promter, P*rep* activity on the initial fragment was strong. Consequently, the fragment containing nt −354 to +1 was truncated from the 5′ end. The highest activity was observed with plasmid pGL-194 (nt −193 to −1). A substantial decrease in Luc activity was obtained when the 5′ end of the fragment carrying nt −193 to −1 was further truncated to nt -105, indicating that the three SP1 sites before ISRE of P*rep* must be important for the complete activity of P*rep*. Interestingly, the pGL-108 fragment carrying nt −193 to −85 also shows transcription activity, even it was lower compared to pGL-194 or pGL-106 (Figure 2).

From the promoter activity assay we found that the promoter containing three tandem sp-1 sites and the ISRE sequence with highest activity. P*rep* is composed of two independent mini promoters, the proximal one containing the ISRE sequence spans the sequence +1 to −106, while the distal mini promoter is composed of three sp-1 sites.

### The ISRE is essential for porcine IFN-α induced *Rep* promoter enhancement

**Figure 3:**
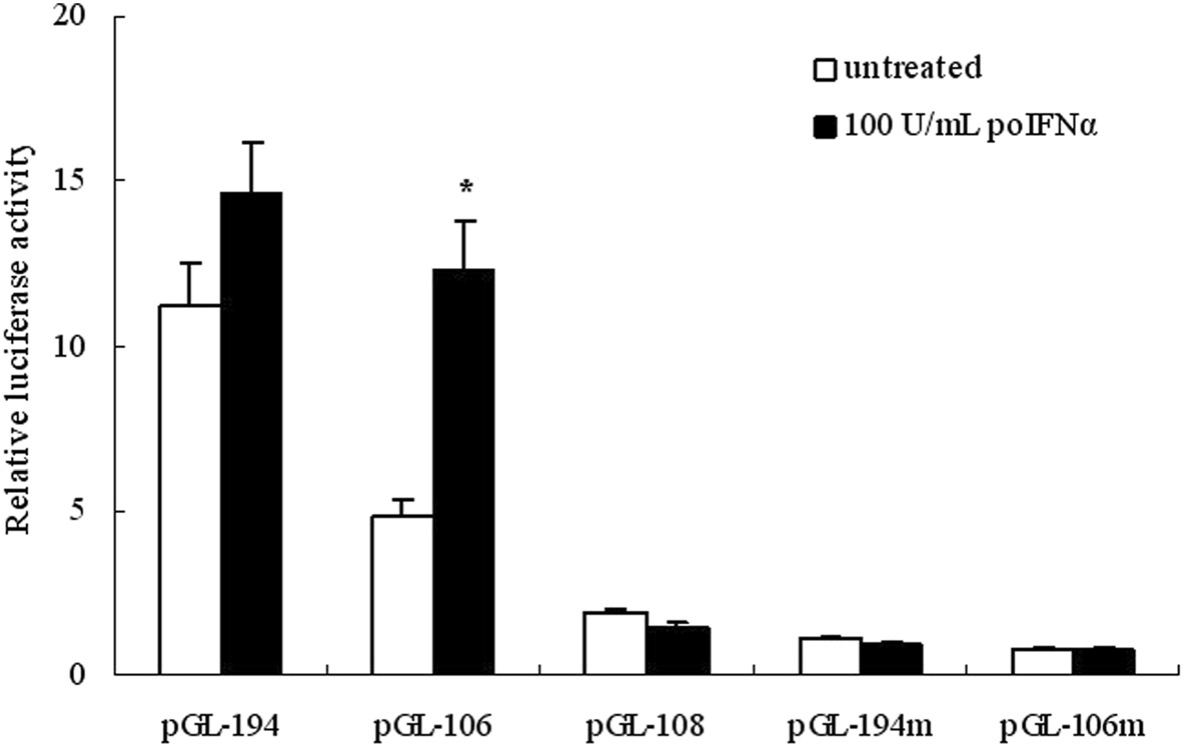
** Response character of different constructs of porcine* Rep* promoter to IFN-α treatment.** The figure shows the transcription activities of serials constructs assayed in PK-15 cells with or without induction of 100 U/mL IFN-α. The transcription activity of PGL-106 increased significantly in the presence of IFN-α. Experiments were carried out independently at least 3 times and the results are expressed as means ± SD.

To test whether *Rep* promoter can be activated upon porcine IFN-α treatment, the luciferase reporter plasmids pGL-194, pGL-108 and pGL-106 were transfected into PK-15 cells, and the expression of luciferase, after porcine IFN-α treatment, was measured. As shown in Figure 3, The transcription activity of pGL-194 and pGL-106 increased by 135% and 220% respectively in the presence of 100 U/mL porcine IFN-α. However, pGL-108 which contains no ISRE, transcription activity decreased by 25% in the transfected cells treated with porcine IFN-α. To determine whether viral ISRE is necessary for porcine IFN-α initiated luciferase expression enhancement. The plasmids designated pGL-194 m and pGL-106 m were constructed by replacing the viral ISRE sequence 5′-CTGAAAACGAAAGA-3′ with the mutant sequence 5′-CTcAAtACcAAAGA-3′. When the two plasmids were transfected into PK-15 cells, porcine IFN-α no longer were able to enhance luciferase reporter gene transcription. This result strongly suggests that the viral ISRE is essential for IFN-α-initiated enhancement of PCV2 *Rep* promoter. However, mutation of the ISRE site decreased *Rep* promoter basal activity eight to ten fold. The Luc activity obtained with the mutated ISRE *Rep* promoter was even lower than that obtained after deletion of the complete ISRE region, suggesting that the transcription activity of tandem SP-1 elements in *Rep* promoter may be affected by the ISRE.

### ISRE Mutation affects replication efficiency and IFN-α response of PCV2 in PK-15 cells

To determine if there is a relationship between ISRE mutation and sensitivity of PCV2 to IFN-α *in vitro*, ISRE mutations were introduced by site-directed mutagenesis and the genomes of wild-type and mutant PCV2 were transiently transferred into PK-15 cells by transfast transfection. Both the wild-type PCV2 genome and the ISRE mutant PCV2 genome produced infectious progeny PCV2 as identified by IPMA. After three cell passages and confirmation by immunochemical staining virus infected cell DNAs were isolated and amplified by PCR with PCV2-specific primers PCV2 F3 and PCV2 R2. After Gel purification each PCR product was sequenced. The result showed that the engineered mutation was retained in the progeny viruses. The two recovered PCV2 were both genetically stability during 3 passages in PK-15 cells.

**Figure 4:**
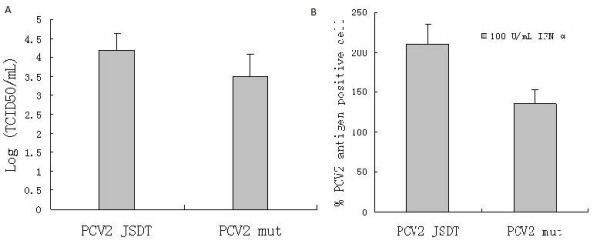
**A. PCV2 JSDT and PCV2 mut titers in PK-15 cells.** Experiments were carried out independently at least 3 times and the results are shown as means ± SD. **B.** Effects of porcine IFN- α on the number of PCV2 antigen positive PK-15 cells. PK-15 cells were treated with 100 U/mL IFN-α at 1 hours post virus inoculation. The number of PCV2 antigen positive cells in IFN-α treated cells were counted after 36 h incubation and compared with the number of PCV2 antigen positive cells in untreated cells. Experiments were carried out independently at least 3 times and the results are expressed as means ± SD.

In contrast to wildtype PCV2, the ISRE mutant exhibited weaker replication efficiency. The virus titer of wildtype PCV2 and the mutant PCV2 were about 10^4.19^ TCID_50_/mL and 10^3.50^TCID_50_/mL respectively (Figure 4A). After 100 U/mL porcine IFN-α was added, viral antigen positive cells of both the wild-type PCV2 and the ISRE mutant PCV2 increased, up to 210% and 135% respectively (Figure 4B). These data indicate that PCV2 harboring the ISRE mutation has weaker replication efficience and lower sensitivity to IFN-α than wild-type.

### PCV2-specific serum antibody responses

**Figure 5:**
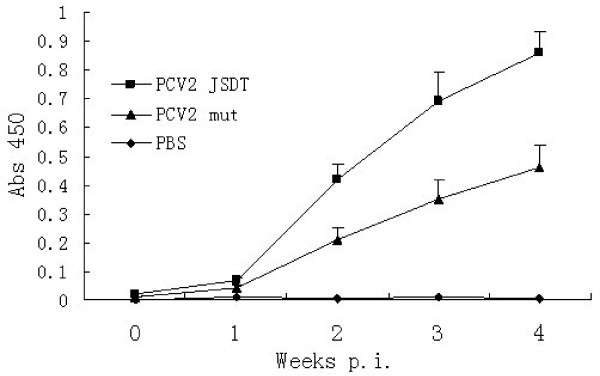
**The kinetics of PCV2-specific IgG antibody production at various times p.i.** Experiments were carried out independently at least 3 times and the results are shown as means ± SD.

The production of PCV2-specific IgG antibodies by each of the animal groups was monitored by PCV2 antibody ELISA kit after inoculation. The final result is expressed as a signal: positive (S:P) ratio. As shown in Figure 5, PCV2 infection induced IgG after 1 week p.i. piglets inoculated with PCV2 JSDT exhibited higher PCV2-specific IgG titers with S:P ratios of 0.42 ± 0.05, 0.69 ± 0.1, and 0.86 ± 0.07 at 2, 3 and 4 weeks p.i., respectively, compared to PCV2 mut titers of 0.21 ± 0.04, 0.35 ± 0.07, and 0.46 ± 0.08 at the same time points, respectively, these differences were statistically significant (*P* < 0.05). These data clearly showed that the wildtype PCV2 infection elicited a higher antibody response than the ISRE mutant PCV2 infection.

### Quantification of PCV2 in serum from infected piglets

**Figure 6:**
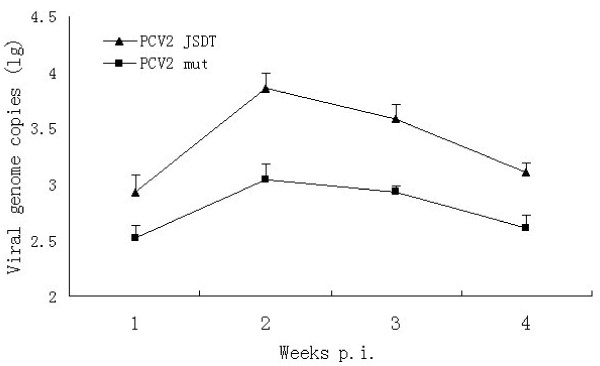
**The viremia of wildtype or ISRE mutant PCV2 infected piglets.** Experiments were carried out independently at least 3 times and the results are shown as means ± SD.

To identify whether mutating the ISRE sequence affect viral replication *in vivo*, the PCV2 genomic copy numbers in serum were quanficated by real-time PCR and the data were presented as the log_10_ value of PCV2 viral genomic copy numbers. As shown in Figure 6, viral genomic copies peaked at 2 weeks p.i. and the levels of PCV2 JSDT viremia were higher than the levels of PCV2 mut from 1 to 4 weeks p.i. The results demonstrated that the replication efficiency of ISRE mutant PCV2 was lower than the wildtype PCV2’s *in vivo*.

## Discussion

An ISRE sequence 5′-CTGAAAACGAAAGA-3′ which is precisely conserved among the different gene-types of PCV2 locates at nt 1737–1751 in the PCV2 *Rep* promoter, Previous study [16] found that when the PCV2 ISRE sequence present in the context of intact virus but not in isolation influences the interferon-mediated enhancement of PCV2 replication *in vitro*, they also found that mutating the ISRE sequence did not significantly affect viral replication *in vitro*, but our research demonstrated that PCV2 ISRE sequence is a functional motif in and out of the whole viral genome and it can affect the PCV2 replication *in vitro* and *in vivo*.

Porcine IFN-α could enhance the transcription of PCV2 ISRE reporter construct significantly. Even though the absolute lucifease expression of all reporter constructs include pGL3-promoter were significantly decreased in the presence of IFN-α treatment due to systemic inhibition effect of translation by IFN-α on host cell gene (data not shown). The relative lucifease activity (firefly luciferase/Renila luciferase) of the pGm-PCV2-ISRE (3) increased by two fold in the presence of 100 U/mL porcine IFN-α. The findings are somewhat different from the results by S. Ramamoorthy et al [16], they concluded IFN-α or IFN-γ treatments of 3D4/31 cells transfected with the reporter gene constructs carrying the wildtype ISRE sequence did not increase the reporter gene expression. It is possible that the cells, vectors and the rep promoter gene fragments we used were different. They used 3D4/31 cells and pGL3-basic vectors, whereas we used PK-15 cells and pGL-mini vectors, the gene fragments they used were relatively intact rep promoter, but what we used were only three copies of ISRE in this part.

The obvious functional experiment was to test whether Rep promoter can be activated upon IFN treatment. However, the full-length Rep promoter reporter construct did not respond to porcine IFN-α treatment significantly. This result might be due to negative regulatory elements in the promoter or the basal level of luciferase activity was high after transfection. For the transfection procedure itself activated interferon expression(data not shown). Interferon deficiency cell line suitable for porcine circovirus is now not available. In order to solve this problem, Rep promoter was further characterized.

Computer-assisted sequence analysis showed that PCV2 *Rep* promoter does not contain any recognizable TATA or CAAT elements. However, besides a sequence, spanning nt -80 to nt -67 that is homologous to the ISRE present in the promoter of ISGs, there are GC box like sequences at four different sites before *Rep* gene, which are known to implicate in transcription.

Interestingly, from the promoter activity assay using various promoter constructs, we found that promoter construct lacking GC box at −192 to −84 significantly reduced luciferase activity, suggesting that *Rep* expression might be partially controlled by a GC box binding protein such as transcription factor Sp-1 [18,19]. Sp1 is a ubiquitous transcription factor which is responsible for activating many cellular and viral genes. Like PCV2 *Rep*, many eukaryotic genes with TATA-less promoters contain multiple Sp1-binding motifs arranged in tandem and in close proximity within the proximal promoter [20]. Sp1 can activate many of these promoters synergistically by Sp1–Sp1 interactions and these interactions are an important mechanism for modulating the expression of this class of genes [21].

Further study indicated that P*rep* is composed of two independent mini promoters, the two mini promoters are both able to initiate the expression of luciferase gene and GFP gene in PK-15 cells (pictures not shown). With the proximal *Rep* promoter, mutation of the ISRE was shown to abrogate IFN-α mediated up-regulation of PCV2 proximal *Rep* promoter driven reporter gene expression. This suggested a crucial role of the ISRE in IFN-α induced enhancement transcription. Based on the responsiveness to IFN-α, and the viral ISRE sequence similarity to consensus ISRE, we concluded that *Rep* promoter has a functional ISRE sequence. With the distal Rep promoter, decreased luciferase transcription was observed in the presence porcine IFN-α treatment. There are three GC box like sequences which predicted to be SP-1 binding site in the computer-assisted scanning. Within the Sp-family transcription factors, Sp-1 and Sp-3 are ubiquitously expressed in mammalian cells. Sp-3 is structurally similar to Sp-1, with similar affinities for the Sp-1-binding site [21]. Previous study suggested different roles for Sp-1 and Sp-3, with Sp-1 being involved in basal transcription and Sp-3 having a role in the interferon inducible transcription of the PKR gene [22]. Whether GC box like sequence is the binding site of Sp-1 or Sp-3 and contribute to the regulation of Rep promoter has yet to be determined.

The luciferase transcription activity of the full-length *Rep* promoter is higher than the value of distal *Rep* promoter with proximal *Rep* promoter, so we conclude the two mini-promoters have a synergistic effect. However, luciferase transcription activity of the ISRE mutant full-length *Rep* promoter is even lower than the distal *Rep* promoter, indicating the PCV2 ISRE sequence plays an important role in the activity of *Rep* promoter.

PCV2 *Rep* gene promoter is known to support the synthesis of *Rep* and *Rep*’, *Rep*’ is truncated, and the C terminus is expressed in a different reading frame. Two products of the *Rep* gene are indispensable for PCV replication [23]. The *Rep*-complex recognizes and binds the direct H tandem repeats and the right-arm of the palindrome destabilizes and unwinds the Ori sequence, and then nicks the octanucleotide sequence between T6 and A7 to generate a free 3′-OH end for initiation of plus-strand DNA replication [24]. Besides its role in replication, the *Rep* protein is a transcriptional repressor of *Rep* gene expression. Previous study indicated that the full-length *Rep* protein could repression the P*rep* by binding to the two inner hexamers, H1 and H2, located in the origin of PCV1 [25]. So the transcriptional activity of P*rep* is controlled by both viral and cellular factors. Since the *Rep* and *Rep*’ are essential for PCV DNA replication, their expression regulation is expected to affect progeny virus production.

The results of viral mutational analysis *in vitro* demonstrated that the ISRE is non-essential for virus replication, for the progeny PCV2mut with function abrogated ISRE was readily recovered from cultures transfected with the excised and recircularized double-stranded mutant genomes. However, PCV2mut decreased replication competence and lower sensitivity to IFN-α were observed demonstrated a significant role of ISRE in the viral replication efficiency and regulation of IFN-α-mediated PCV2 replication on PK-15 cells.

The data of animal experiments showed that the mutation of PCV2 ISRE sequence not only affect the replication efficiency in PK-15 cells, but also had negative effects *in vivo*, the ISRE mutant PCV2 infection elicited a lower antibody response and replication efficiency than the wild type PCV2 infection. Therefore, we conclude that the PCV2 ISRE is an important motif in the viral transcription start *in vivo* and *in vitro.*

The interaction of viral ISRE located in the *Rep* promoter of PCV2 with host cell transcription factor regulated by IFN may be anther underlying mechanism for enhanced virus production. Previous study has reported that up-regulation of PCV2 in PK-15 cells by IFN-γ is due to enhanced internalizing of viral particles [3]. Besides ISGF3, IRFs are another important factor induced by type I and II IFNs. Since the sequence of IRF binding site termed IRF-E overlaps with ISRE, IRF also binds to ISRE sequence and activates the interferon-inducible gene transcription, but in part, functionally different [9]. So which transcription factor binds the PCV2 ISRE? Further research should clarify this issue.

## Conclusions

From all of the research results, we conclude that PCV2 ISRE is an unquestionable functional element to IFN response; it also plays an important role in viral transcription start. The findings provide a theoretical basis for the Phenomenon of IFNs are able to enhance PCV2 infection and production in PK-15, they also improve the understanding of interaction of PCV2 with their host cells, the pathogenesis and reproduction of PMWS in PCV2-infected pigs.

## Materials and methods

### Cell, virus and serum

PK-15 cells free of PCVs were grown at 37°C in a humidified 95% air–5% CO_2_ atmosphere in minimal essential medium (MEM) (GIBCO-BRL, Grand Island, NY) supplemented with 10% fetal bovine serum, 50 U of penicillin per mL, 50 μg of streptomycin per mL, and 2 mM glutamine. The PCV2 strain JSDT (accession number GQ227412) used in the study was originally isolated from Lymph nodes sample of a pig with naturally occurring PMWS. This virus was propagated in PK-15 cells and confirmed to be PCV2 by PCR analysis and reactivity with specific PCV2 monoclonal antibodies. The hyper-immune swine serum against PCV2 was collected from clinical serum identified by ELISA.

### Recombinant porcine interferon alpha

Porcine IFN-α were produced in *Pichia Pastoris* yeast expression system and the activity were measured using bioassay described previously [26]. The IFN was diluted just before use in MEM supplemented with 10% FBS to the final concentration used in the assay.

### Promoter scan

The presence of putative regulatory elements in the promoter sequences was analysed online with Proscan: Version 1.7.

### DNA mutagenesis

The complete PCV2 genome was obtained by PCR and was then cloned into pUC18 digested with *EcoR* I to generate pUC18-PCV2. This plasmid was used as a substrate for the mutagenesis of the ISRE site. A pair of primers namely PCV2mF and PCV2mR (Table 2) were designed. The genome mutation was accomplished by using the QuikChange Site-Directed Mutagenesis Kit (Stratagene), according to the manufacturer’s instructions. The mutation product was verified by gene sequencing.

**Table 2:** Oligonucleotide primers used in this study

**Primer**	**Primer sequence**	**Application**
PCV2 F1	5′-GCGAATTCAACCTTAACCTTTCTTATTC-3′	PCV2 genome clone
PCV2 R1	5′-ATGAATTCTGGCCCTGCTCCC-3′	
PCV2 mF	5′-CAGCGCACTTCTTT**g**GT**a**TT**g**AGATATGACG-3′	ISRE mutation
PCV2 mR	5′-CGTCATATCT**c**AA**t**AC**c**AAAGAAGTGCGCTG-3′	
PCV2 F2	5′-CATGGTACCAGAGCGGGGGTTTGA-3′	pGL-355 construction
PCV2 R2	5′-GCTAAGCTTGTTGCTGCTGAGGT-3′	
PCV2 F2	5′-CATGGTACCAGAGCGGGGGTTTGA-3′	pGL-270 construction
PCV2 R3	5′-CAGAAGCTTTGACGTATCCAAGAAGGC -3′	
PCV2 F3	5′-CCTTCTCCAGCGGTACCGGT-3′	pGL-194 and pGL-194 m construction
PCV2 R2	5′-GCTAAGCTTGTTGCTGCTGAGGT-3′	
PCV2 F3	5′-CCTTCTCCAGCGGTACCGGT-3′	pGL-108 construction
PCV2 R3	5′-CAGAAGCTTTGACGTATCCAAGAAGGC -3′	
PCV2 F4	5′-CATGGTACCAACGCCTTCTTGGAT-3′	pGL-106 and pGL-106 m construction
PCV2 R2	5′-GCTAAGCTTGTTGCTGCTGAGGT-3′	

Mutated nucleotides in the PCV2 ISRE are indicated in lower bald case.

### Plasmid construction

The enhancer test vector pGL-miniP was constructed by substituted the SV40 promoter of pGL3-Promoter with HSV-1 TATA-like promoter (P_TAL_). pGm-PCV2-ISRE(3) and pGm-PCV2-ISREmut(3) were made by inserting three copies of PCV2-ISRE and PCV2-ISREmut double oligonucleotides into the unique *Xho* I site of pGL-miniP. The oligonucleotide pairs used to generate the PCV2-ISRE and PCV2-ISREmut double oligonucleotides were TCGAGCTGAAAACGAAAGAAGTGC, TCGAGCACTTCTTTCGTTTTCAGC (oligo PCV2-ISRE, PCV2 nucleotide coordinates 1736–1757) and TCGAGCTcAAtACcAAAGAAGTGC, TCGAGCACTTCTTTgGTaTTgAGC (oligo PCV2-ISREmut, mutated nucleotides in the PCV2 ISRE are indicated in lower case), respectively.

To character the PCV2 *Rep* gene promoter, the following plasmids were used in this study. DNA fragments for cloning were PCR-generated with PCV2 JSDT as the template and the primers used were listed in Table 1. pGL serial plasmids carry fragments of the putative P*rep* promoter. Plasmid pGL-355 carries the PCR-derived and *Kpn* I/*Hind* III tagged fragment nt −354 to +1 of PCV2 *Rep* gene cloned into the *Kpn* I/*Hind* III restricted vector pGL3-basic. pGL-194 carries the fragment nt −193 to +1; pGL-270 nt −354 to -84; pGL-108 nt −192 to −84; pGL-106 nt −105 to +1. To introduce the ISRE mutations in a luciferase reporter system, PCR was used to amplify the promoter region from the mutant viral genome described above. While pGL-194 m and pGL-106 m carry the PCR-derived fragments from ISRE mutated PCV2 genome respectively. All plasmids have been sequenced to exclude PCR acquired mis-incorporation of nucleotides. Plasmid pGL3-control was used as a positive control and plasmid pGL3-basic as a negative control (Promega).

### luciferase reporter assays

PK-15 cells grown overnight in 24-well plates were transfected 1 μg of reporter plasmids and 0.25 μg of *Renilla* luciferase plasmid (pRL-TK [Promega]) with Transfast reagent (Promega). At 36 h post-transfection, the cells were harvested and washed twice with phosphate-buffered saline, and luciferase enzyme activity was measured by using the dual-luciferase assay system (Promega). For these assays which detected the response to IFN-α, the transfection mixture was removed and replaced with media containing 100U/mL porcine IFN-α at 12 h post-transfection. Relative luciferase activity was normalized to the activity of *Renilla* luciferase used as an internal control. Representative data of three experiments done in triplicate are shown.

### Transfection of PCV2 genomes and identification of progeny virus

After the viral genomes were excised from pUC18-PCV2 or pUC18-PCV2m and re-circularized by T4 DNA ligase, the ligated DNA mixture (2 μg) was used to transfect 60 to 80% confluent monolayers of PK-15 cells which had been seeded into 24-well tissue culture plates 24 h later using Transfast reagent (Promega). After transfection at 37°C for 72 h, the transfected cell cultures were frozen and thawed thrice to make a virus stock. The viability of the wildtype virus and the mutant virus stocks were subsequently determined by infecting PK-15 cell monolayers with a 1:10 serial dilution of the two virus stocks and were detected by the method of immunoperoxidase monolayer assay (IPMA) which is described elsewhere [27]. At the meantime, to identify genetic stability of the wildtype virus and the mutant virus stocks, the progeny virus was passaged in PK-15 cells for three times, the *Rep* promoter region of 3th progeny virus was amplified using preimers PCV2 F3 and PCV2 R2, the PCR products were then gene sequenced. The 3th progeny wildtype virus PCV2 JSDT and the mutant virus PCV2 mut were used to analysis the replication efficiency and the response to IFN-α treatment in PK-15 cells.

### Analysis the replication efficiency and the response to IFN-α treatment in PK-15 cells

To compare the replication efficiency of the wildtype virus PCV2 JSDT and the mutant virus PCV2mut, the two viruses should be adjusted to the same multiplicity of infection (moi) of 1 to infect the 60 to 80% confluent monolayers of PK-15 cells which had been seeded into 6-well tissue culture plates containing 1 × 10^9^ cells/well, 72 h later, the plates were frozen and thawed thrice and the virus stock was titrated again in PK-15 cells using serial 10-fold dilutions of each sample.

To analysis if PCV2 harboring the ISRE mutation can reduce the sensitivity to IFN-α, the 96-well plate containing 1.5 × 10^4^ cells/well was used in this experiment, PK-15 cells were inoculated when they were 60 to 80% confluent (24 h after seeding). The wildtype virus PCV2 JSDT and the mutant virus PCV2 mut infected PK-15 at the same multiplicity of infection (moi) of 0.5, 1 h after inoculation, the inoculum was replaced with new culture medium and one half of infected cells treated with 100U/mL IFN-α while the other half left untreated. After 36 h incubation, the cells were dried and frozen at -20°C. The plates were stained using an immunoperoxidase monolayer assay (IPMA) and the number of PCV2 antigen-positive cells was counted by light microscopy.

### Animal experiment

A total of 24 3-week-old piglets without antibodies against PCV2 were purchased from a herd, all animal procedures were in accordance with the Guidelines for the Care and Use of Animals at Nanjing Agricultural University, The license number is SYXK (苏) 2010–0005. they were randomly divided into three groups (8 piglets per group) that were kept apart in separate isolated rooms. Group 1 piglets were each inoculated with PCV2 JSDT (10^4.0^ TCID_50_/mL, 3 mL intranasally and 3 mL intramuscularly), Group 2 piglets were each inoculated with PCV2 mut (10^4.0^ TCID_50_/mL, 3 mL intranasally and 3 mL intramuscularly), Group 3 piglets served as uninfected control, each received corresponding volumes of Dulbecco’s phosphate buffered saline (Invitrogen) (PBS). Serum samples taken for antibody detection and PCV2 genomic copy numbers analisis were collected prior to inoculation and every week for 4 weeks. PCV2-specific IgG antibody were detected using the ELISA kit (JBT) following manufacturer’s instructions. DNAs were extracted from serum samples using the virus DNA extraction kit (Sangon biotech), the extracted DNAs were used for quantification of serum PCV2 genomic DNA copy numbers by real-time PCR as described [28].

### Statistical analysis

Results are presented as averages ± standard deviations of the means. Statistical comparisons are made by using Student’s *t* test, and differences between groups were considered significant if the *P* value was < 0.05.

## Abbreviations

PCV2: Porcine circovirus type 2; PMWS: Post-weaning multi-systemic wasting syndrome; IFN-α: Interferon-α; PK-15: Porcine kidney-15; ISRE: Interferon-stimulated response element; P*rep*: *Rep* gene promoter; RF: Replication form; ISGF3: Interferon-stimulated gene factor 3; STAT1 and STAT2: Signal transducers and activators of transcription 1 and 2; EBV: Epstein-Barr virus; moi: Multiplicity of infection; IPMA: Immunoperoxidase monolayer assay; PBS: Phosphate buffered saline.

## Competing interests

The authors declare that they have no competing interests.

## Authors’ contributions

GJY carried out the molecular genetic studies, participated in the sequence alignment and drafted the manuscript. ZY, LX and SHL participated in the luciferase reporter assays. WJM, LWT, MG, LP, ZD and JYX carried out the immunoassays. GJY participated in the design of the study and performed the statistical analysis. CRB conceived of the study, and participated in its design and coordination and helped to draft the manuscript. All authors read and approved the final manuscript.
